# Barley Melanoidins: Key Dietary Compounds With Potential Health Benefits

**DOI:** 10.3389/fnut.2021.708194

**Published:** 2021-09-28

**Authors:** Jitendra Kumar Sharma, Monika Sihmar, Anita Rani Santal, Louis Prager, Franck Carbonero, Nater Pal Singh

**Affiliations:** ^1^Centre for Biotechnology, Maharshi Dayanand University, Rohtak, India; ^2^Department of Microbiology, Maharshi Dayanand University, Rohtak, India; ^3^Department of Crop and Soil Science, College of Agricultural, Human, and Natural Resource Sciences, Washington State University, Pullman, WA, United States; ^4^Department of Nutrition and Exercise Physiology, Elson Floyd College of Medicine, Washington State University, Spokane, WA, United States

**Keywords:** barley, melanoidins, health benefits, gut microbiome, antioxidant

## Abstract

This paper is a review of the potential health benefits of barley melanoidins. Food melanoidins are still rather understudied, despite their potential antioxidant, antimicrobial, and prebiotic properties. Free radicals are villainous substances in humans produced as metabolic byproducts and causing cancers and cardiovascular diseases, and the melanoidins alleviate the effects of these free radicals. Malt is produced from cereal grains such as barley, wheat, and maize, and barley is predominantly used in beer production. Beer (alcoholic and non-alcoholic) is a widely consumed beverage worldwide and a good source of dietary melanoidins, which enhance the beers' flavor, texture, and sensorial properties. Melanoidins, the final products of the Maillard reaction, are produced at different stages during the brewing process. Beer melanoidins protect the cells from oxidative damage of DNA. The high reducing capacity of melanoidins can induce hydroxyl radicals from H_2_O_2_ in the presence of ferric ion (Fe^3+^). Melanoidins inhibit lipid peroxidation during digestion due to their chelating metal property. However, lower digestibility of melanoidins leads to less availability to the organisms but is considered to function as dietary fiber that can be metabolized by the lower gut microbiota and possibly incur prebiotic properties. Melanoidins promote the growth of Lactobacilli and Bifidobacteria in the gastrointestinal tract, preventing the colonization of potential pathogens. Barley is already popular through beer production and increasingly as a functional food. Considering this economic and industrial importance, more research to explore the chemical properties of barley melanoidins and corresponding health benefits as barley is warranted.

## Introduction

Barley is the fourth most important cereal worldwide, used as human food and animal feed, and the most common raw material in the brewing industry. It is rich in soluble dietary fiber, specifically beta-glucan-like oats. Potential health benefits of barley have been reported, including against diabetes, hypertension, obesity, and colon inflammation (1–3). The health benefits of barley grains can be enhanced by heat treatment of barley grains during their processing. Heat treatment facilitates the formation of melanoidins (4, 5). Melanoidins are brown-colored, polymeric high molecular weight (HMW) compounds found in thermally processed foods and are the end product of the Maillard reaction (MR) (6). The MR, a non-enzymatic browning reaction, occurs between reducing sugars and free amino acids and peptides at high temperatures and produces melanoidins (7, 8). Usually, food or food products are processed at high temperatures for cooking, baking, roasting, frying, or even sterilization. The temperature ranged from 90 to 220°C. At this high temperature, MR, a Non-enzymatic browning reaction, occurs (9). In the formation of melanoidins, a brown nitrogenous polymer and copolymer, an array of reactions include cyclizations, dehydrations, retro aldolization, rearrangements, isomerizations, and condensations take place (9). The chemical structure of barley melanoidins is very complex and largely unknown because polymerization of complex melanoidins is influenced by various factors such as starting materials and their concentration, reaction conditions, such as pH, water activity, temperature, and reaction time, and solvent used (10–12). In order to predict the chemical structure of melanoidins, some recommendations are listed in Table 1.

**Table 1:** The proposed chemical structures of melanoidins.

**Melanoidins structure**	**References**
Melanoidins formed by polycondensation reaction of units of furan and pyrroles.	(13)
Skeleton of melanoidins formed at an early stage of MR by sugar degradation, then polymerization and linked by amino compounds.	(14)
Crosslink between free amino groups of lysine or arginine in proteins.	(15)
Skeleton of melanoidins formed by crosslinking of MR products and proteins.	(16)

Melanoidins are classified based on their skeleton composition. The skeleton of melanosaccharides is composed of polysaccharides, whereas melanoproteins are composed of proteins (17, 18). Melanosaccharides compounds are negatively charged, readily soluble in water developed from amino acids and polysaccharides (19). Melanoproteins have resulted when proteins and sugars of protein-rich food are cross-linking with each other. Melanoproteins are usually water-insoluble and contain HMW molecules (18).

In beverages like coffee and beer, melanosaccharides are the predominant melanoidins, whereas melanoproteins are predominant in bakery products like bread and biscuits (20). Since melanoidins are complex and diverse molecules, they are poorly soluble in water and organic solvents, making it hard to define their specific properties (21). However, the properties of melanoidins are greatly influenced by the low molecular weight (LMW) compounds (<10 kDa), which polymerize during MR and form HMW melanoidins (>300 kDa) (22). Also, the properties of melanoidins depend on the time and temperature applied during the brewing process (23).

**Figure 1:**
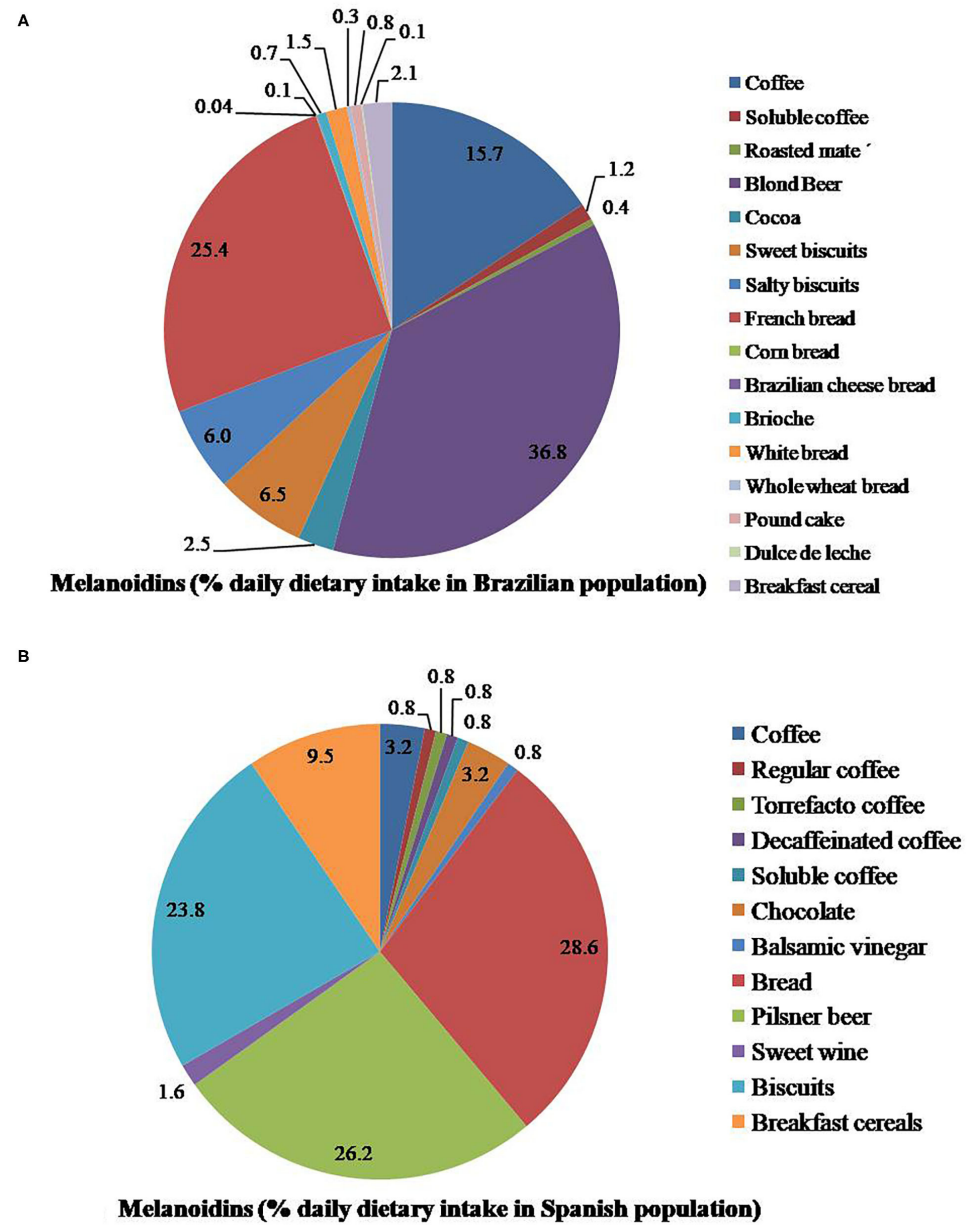
Percentage source of dietary consumption of melanoidins in the **(A)** Brazilian population, **(B)** Spanish population (18, 19).

Several studies were conducted to investigate the health benefits, daily dietary intake of melanoidins in different populations. For example, Papetti et al. (5) investigated the antioxidant activity of melanoidins isolated from roasted barley solution/barley coffee and reported that HMW melanoidins are associated with antioxidant activity. Daily dietary intake of melanoidins in different populations varies (Figure 1). For example, in the Spanish population, the daily dietary intake of melanoidins is 12.2 g/person/day. Therefore, it contributes 20% of the total antioxidant capacity intake, while in the Brazilian population, the daily dietary intake of melanoidins is 10.7 g/person/day and contributes around 21% of dietary antioxidant capacity intake (18, 19).

**Figure 2:**
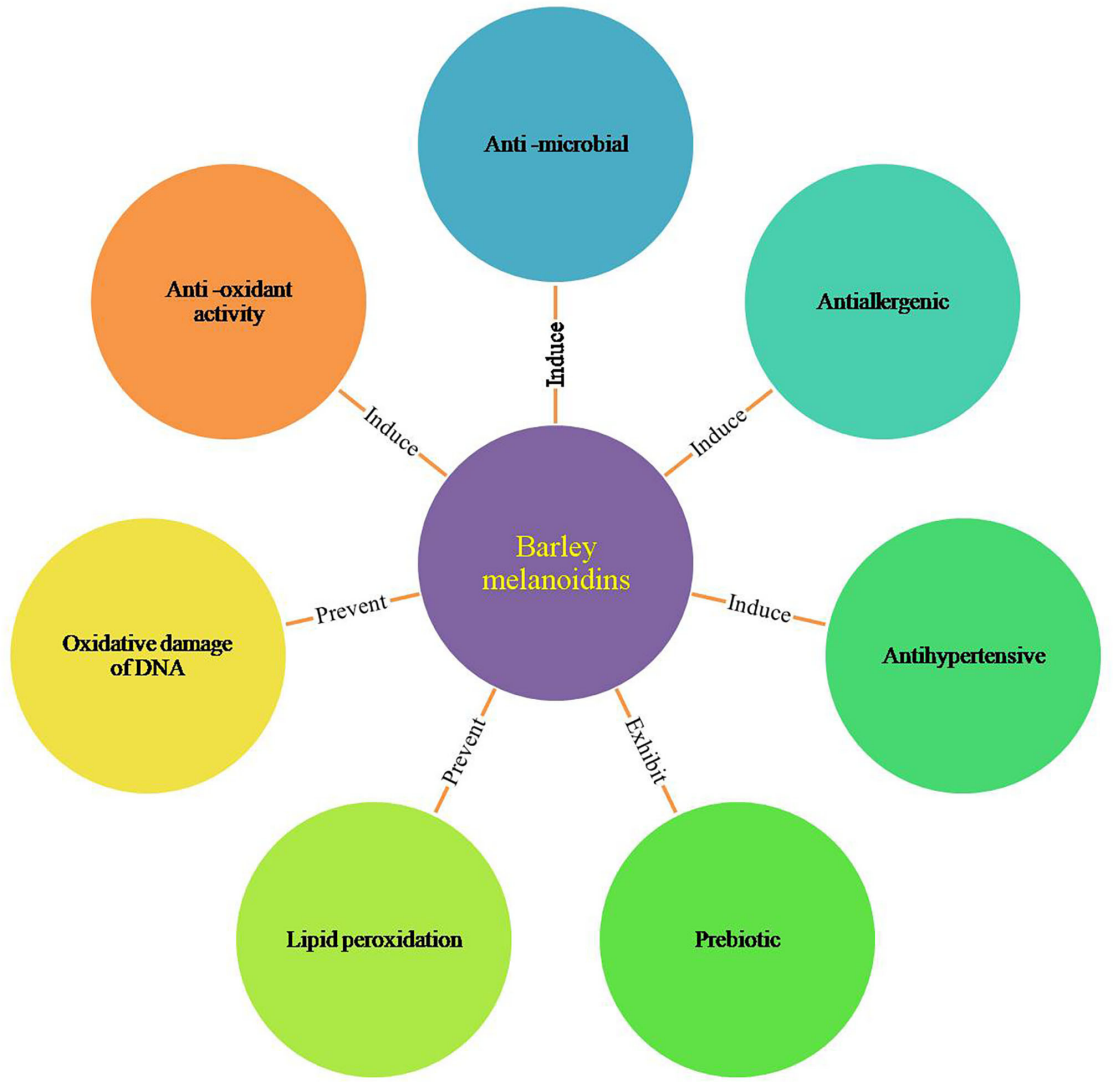
Major biological activities of barley melanoidins.

Several studies reported that melanoidins prevent lipid peroxidation, oxidative damage of DNA, and have antimicrobial, antihypertensive, antiallergenic, and prebiotic properties (Figure 2) (9, 24). The present review will focus on the health benefits of melanoidins, especially from barley sources. It is very important to conduct more research to explore the chemical properties of barley melanoidins and their corresponding health properties as barley has economic and industrial importance.

## Barley Sources of Melanoidins

### Barley Malt

Based on color, barley malts can be categorized into pale and dark malt. In the production of beer, pale malt is commonly used as the main ingredient. Malting of barley grains is achieved in three steps, steeping, germination, and kilning. The malting process begins with steeping raw grain underwater to bring moisture content up from 12 to (42–46%). The first steeping lasts between 6 and 16 h and brings the grain up to 33–37% moisture content. Next, the water is removed from the grain bed, removing any moisture film and carbon dioxide produced during respiration. The grain then goes through an air rest for 12–24 h, exposing the embryo to oxygen. Next, the grain is immersed in the water again for 10–20 h to bring the final moisture content of 42–46% (25, 26). After steeping, the hydrated grain or green malt is stored in a well-ventilated humid area kept between 14 and 20°C. Enzymes are activated and begin degrading cell walls, proteins, and starches. The green malt is kept in this germination stage for 4–6 days, during which some moisture is lost and replaced by spraying the green malt with water. Germination is measured by the rootlets that begin to grow, and once the rootlets are between 1.5 and 2 times, the germination period is complete (25, 26).

Kilning or roasting is the final step in the malting process. Here the green malt is thermally processed, drying the malt to a moisture content of ~5%. This step inactivates and preserves the enzymes and ensures stability for the storage and transportation of the finished malt. The first phase is referred to as the “whitering” or “free drying” phase. Next, the grain is air-dried at 25°C, decreasing the moisture content from 44 to 12%. The next phase is a much longer process taking the malt from 12% moisture content to 4%. This is referred to as the “falling rate phase.” After the grain is dried, the final step is an increase in temperature, initiating the “curing stage” followed by a cooling period and finalized with the packaging of the final product (25, 26). It is important to note that the kilning and roasting step contributes the most to the color and flavor profiles of the malted barley (27). Dark malt is further classified into color brew malts, caramel malts, and roasted malts, and their production is dependent on the temperature on which they are produced. Malt produced from roasted barley can be classified into Pale malt (products include amber, chocolate, and black malt), green malt (products include cara, crystal, dark crystal, caramel malt), and colored kilned malts (includes Munich malt and Vienna malt) (26). Temperature up to 105°C is used to produce color brew malt, while caramel malts and roasted malts are up to 160 and 220–250°C for 2–2.5 h, respectively (28).

### Beer Melanoidins

Various types of beers are produced and consumed worldwide. They differ from each other in color, flavor, and alcohol content, including ale, lager, porter, stout, blonde ale, brown ale, pale ale, India pale ale pilsner, etc. Melanoidins influenced beer characteristics, such as color, flavor, and body of the beer despite having positive effects on health (23). The type of beers melanoidins influence content in the beer and ranges between 0.06 and 10.3 g/100 ml (29, 30).

The quantification of melanoidins, phenolic compounds and sulfur dioxide, as well as the assessment of antioxidant activity of 40 lager beer was performed by Zhao et al. (30). They reported 8-fold variation in the melanoidins content of beer which ranged from 1.64 to 13.71 g/L. The variation in melanoidins content of different beers occurred due to differences in the raw material used and the brewing process. Rivero et al. (29) compared the melanoidins content of dark, blond, and alcohol-free beer and reported the highest level of melanoidins in dark beer with values of 1.49 g/L than blond beer (0.61 g/L) and alcohol-free beer (0.58 g/L). Tagliazucchi and Verzelloni (31) analyzed dark beer and found melanoidins content of 12.3 g/L.

Beer melanoidins intake is depending on the consumption of beer in the populations and data varying between countries. Beer consumption per capita in different countries is different. In 2011, Ireland was on top in per capita beer consumption per year that was 138 L, and this consumption was 1.7 times greater than consumed by a person per year in the United States, while beer consumed by the Czech Republican population was almost equal to the Ireland population (32). As discussed above, beer melanoidins have positive effects on human health, melanoidins from the dark beer demonstrated antioxidant activity and may be used to prevent oxidative damage. In addition, dark beer melanoidins might inhibit lipid peroxidation during gastric digestion and prevent the formation of secondary lipoxidation products, which have adverse health effects, like atherosclerosis (33).

## Health Benefits of Melanoidins

Melanoidins provide a health benefit to human health in various ways. Melanoidins can trap positively charged ion species (electrophiles), scavenge free radicals, and also have reducing power; chelating metal ions lessen oxidative damage (34). Antioxidative capacity of melanoidins can be assayed using DPPH (2,2-diphenyl-1-picryl-hydrazyl-hydrate) assay for free radical scavenging activity ABTS+ (2,2-azinobis-3-ethylbenzothiazoline-6-sulfonate) assay for antioxidant activity, and Ferric Reducing Antioxidant Power (FRAP) assay for ferric reducing ability (21). The occurrence of free ions of iron (Fe^2+^)/ free heme (HmFeIII) groups/heme-containing peptides like metmyoglobin in gastric fluid catalyzes lipid peroxidation (33). Reactive oxygen species (ROS) induces oxidative damage to the DNA, and the melanoidins can counter this harmful effect of ROS (23). Barley melanoidins acting as dietary fiber and increases the growth of beneficial bacteria. Prebiotic properties of melanoidins can be assessed by quantifying Fecal Short-Chain Fatty Acids (SCFAs) (35). Barley sources of melanoidins and their biological activities and assaying methods are listed in Table 2.

**Table 2:** Methodology and source of melanoidins used in the assessment of biological activities.

**Assessment of activity**	**Barley melanoidins source**	**Methodology**	**References**
Lipid peroxidation	Barley coffee and dark beer	Radical-Scavenging Activity by ABTS assay, Lipid Hydroperoxides (LHP) Measurements by FOX assay.	(33)
Antioxidant activity	Beer melanoidins	ABTS radical cation scavenging activity, DPPH radical scavenging activity, Oxygen radical absorbance capacity assay	(30)
Antioxidant activity	Dark and blonde beer	FRAP and TEAC assays	(18)
DNA damage, Antioxidant activity	Lager beers	Folin–Ciocalteus reaction, catechin content by vanillin reaction, DMPD (N, N-dimethyl-p-phenylenediamine) assay	(29)
DNA damage, Antioxidative capacity	Barley flour	DPPH radical scavenging activity.	(36)
Antioxidant activity	Dark beer, barley coffee	Radical scavenging activity- ABTS assay	(31)
Antioxidant activity, antiradical activity	Black beer and pilsner beer	ABTS assay, DPPH assay (antiradical activity), FRAP assay (ferric-reducing ability), Oxygen Radical Absorbance Capacity (ORAC) assay, HOSC assay (hydroxyl radical-scavenging capacity)	(19)
Antioxidant activity	Roasted barley	DPPH Assay, Linoleic Acid-β-Carotene Assay	(5)
Prebiotic potential	Melanoidins-rich barley malts	Fecal SCFAs Quantification	(35)
Antimicrobial activity	Barley coffee	Microtiter plate assay for *Streptococcus mutans* Biofilm	(37)
Antimicrobial activity	Barley coffee	Minimum inhibitory and bactericidal concentration assay for *Streptococcus mutans* Biofilm	(38)
Antimicrobial activity	Pilsner-style beer, Abbeys-style beer, and dry-stout beer	Antimicrobial assay for E. coli and S. aureus	(39)
Antioxidant activity	Brewers' spent grains	DPPH and FR assay	(40)

### Melanoidins as Antioxidants

Maillard reaction products (MRPs), melanoidins, contribute antioxidant capacity to the food products. For example, wheat bread exhibits increased antioxidant capacity by adding barley fiber with wheat flour, and baking enhances the antioxidant property (41). Heat treatment of the malt produces melanoidins and has antioxidant properties, resulting from reducing sugars, and amino acids or proteins (42). The strong reducing capacity of melanoidins proves substantial health-promoting effects (9, 22). Zhao et al. (30) studied the antioxidant activity of lager beer and reported a positive correlation between antioxidant property and melanoidins content (*p* < 0.05), also phenolic content correlated with the antioxidant activity. Morales (43) evaluated the hydroxyl radical scavenging potential of three different European beers: dry-stout beer, abbeys style beer, and pilsener beer. The abbeys-style beer exhibits higher radical scavenging potential than dry-stout beer and pilsener beer.

In India, barley is commonly consumed as “Sattu” prepared by roasting barley grains in sand heated to 250–300°C for a short time, removing the husk, and then ground to flour. Roasting is attributed to the increased antioxidant potential of barley grains and their products. In roasted barley, there are high indicators of antioxidant activity such as metal chelating activity, radical scavenging capacity, and reducing power (44, 45). Carvalho et al. (22) studied the antioxidant potential of three different barley malt produced at different kilning temperatures, 80–85°C for pale malt, 130°C for melano, 80 230°C for black malt. They assayed the antiradical potential and reducing the power of these three barley malt using metmyoglobin assay, deoxyribose assay, and FRAP assay, respectively. High reducing the power of melanoidins can induce conversion of H_2_O_2_ into •OH (hydroxyl radical) in the presence of Fe^3+^ ion. Black malt showed high reducing properties than melano 80 and pale malt. Also, black malt's radical scavenging capacity is higher than melano 80, and pale malt in metmyoglobin assay and deoxyribose assay result is reciprocal of metmyoglobin assay (22). Pastoriza and Rufián-Henares (19) have been investigated the contribution of melanoidins to the antioxidant capacity of the Spanish diet. They reported 8.7 and 15.0 g/100 mL in Pilsen and black beers, respectively (19). Alves et al. (18) studied the various heat-processed food consumed in the Brazilian diet. On average, 10.7 g of melanoidins are consumed daily by this population and contribute up to 21% of the dietary antioxidant capacity of the Brazilian population (18). Antioxidant properties of food items consumed were evaluated using FRAP and TEAC (Trolox Equivalent Antioxidant Capacity) assay. In dark and blonde beer, monosaccharides contents were 4.2 ± 0.6 and 2.0 ± 1.3 g/100 mL, respectively (18). Brewers spent grains (barley) or distilled spent grains (sorghum) are high in moisture and can easily get rotten and cause an environmental problem. However, these byproducts are used in animal feed production or used for biogas production (46). Nonetheless, brewers spent grains or distilled spent grains are rich in melanoidins and established for their potential of antioxidant activities. Spent grains of barley exhibit three times more antioxidant capacity than sorghum spent grain (46, 47). Piggott and coworkers evaluated brewers' antioxidant activity and found greater antioxidant potential in black spent grain than pale spent grain. Ultra filtrate fraction of black spent grain, HMW fraction (>100 kDa) dominate over LMW (< kDa) fraction in antioxidant activity and metal chelating ability, and these properties are attributed by the melanoidins present in HMW fraction (47).

### Melanoidins as an Inhibitor of Lipid Peroxidation

The elevated production of ROS leads to tissue dysfunction and is associated with various pathophysiologies. Lipid peroxidation by ROS produces peroxides and aldehydes. Tissues that are far distant from the site of aldehyde generation are also damaged because these aldehydes are highly stable than parent ROS and can quickly diffuse. The lipid-derived products by ROS are highly reactive and cause noticeable biological effects. Various ailments in humans are caused by these lipid peroxidation products, such as atherosclerosis, reperfusion injury, coronary artery disease, Alzheimer's, Rheumatoid arthritis, and tumors, neurodegenerative diseases, and other disorders. Lipid peroxidation also causes damage to protein and DNA, changes in cell signaling (48–50). Modern-day foods are rich in oxidized and oxidizable lipids, especially fast foods, which elevated lipid hydroperoxides in plasma. Increased plasma lipid hydroperoxides can be integrated into lipoproteins and resulted in lipoprotein oxidation (51, 52). Absorption of malondialdehyde (MDA) and 4-hydroxynonenal in the gastrointestinal tract can cause the pathogenesis of cardiovascular diseases (53).

Various studies and reviews on melanoidins and their anti-lipid peroxidation role are reported. Dietary melanoidins can mitigate the adverse effects of lipid peroxidation products mainly by two strategies (i) melanoidins possibly hinder the lipid peroxidation during digestion of lipid-rich food, and (ii) inhibit the production of secondary lipoxidation products (33). Metal chelating ability of barley coffee, dark beer, and coffee melanoidins proves positive health benefits, chelation of metals during gastric digestion inhibits lipid peroxidation. On the other hand, consumption of red meat renders the heme in circulation, which catalyzes oxidative damage, although melanoidins bind with heme contained in meat and check its absorption in the gastrointestinal tract (33).

### Melanoidins Prevent Oxidative Damage of DNA

Cellular structures and molecules are vulnerable to reactive oxygen species, damaging lipid, protein, or DNA. ROS can cause oxidative damage to the DNA in many ways, such as a base lesion, single-strand breaks, double-strand breaks, cross-linking DNA and protein, and chromosomal breakage rearrangement (29, 54–56). Hydroxyl radical (•OH), generated during the reaction between hydro-peroxides and transition metal ions, is very reactive can assail DNA, proteins, and fatty acids (polyunsaturated) (57). An increased risk of the onset of cancer is highly associated with alcohol consumption. Processing alcohol inside the body generated acetaldehyde, which belongs to group1 carcinogen, which interferes with the DNA repair (58). ROS can attack and modify the guanine base of the DNA into 8-hydroxydeoxyguanosine (8-OHdG). During replication, 8-OHdG is paired with thymine or adenine base instead of cytosine, and eventually, accumulation of this transverse mutation in DNA has a detrimental effect on cells or tissue (59). Rivero et al. (29) signifying the beer melanoidins, which can effectively combat the oxidative stress to the cells. The level of 8-OHdG and DNA damage are negatively correlated with the concentration of melanoidins in beer, especially dark beer (29). However, melanoidins consist of high molecular weight compounds and cannot cross the cellular barrier. Morales et al. (60) suggest that intestinal digestion cleaved melanoidins into an absorbable compound with antioxidant activity.

### Barley Melanoidins and the Gut Microbiome

Dietary Maillard Reaction Products (MRP) has been studied sparsely when it comes to their impact on the gut microbiome and their fate in the lower digestive tract (61), while there is slightly more information published on their precursors, such as the Advanced glycation end products (AGEs) (62–64). Even if their complex and diverse structure make it difficult to draw clear predictions, it is widely thought that a significant portion of dietary melanoidins pass through the upper digestive tract and become available for fermentative metabolism by the gut microbiome (65). This model mostly derives from animal studies. For example, Helou et al. (66) used different bread models and confirmed that a significant amount of dietary melanoidins were excreted in rat's feces. Unlike many other fermentable dietary compounds, very little is known about the microbially derived metabolites from dietary melanoidins. N ε-carboxymethyl-lysine (CML) has been used as a biomarker of dietary and endogenous MRP intake (67). The impact of CML and other MRPs on the growth of different bacterial strains has earlier been studied (68). However, it is not clear if CML can be considered a biomarker of microbiome metabolic activities. There has been no published report on metabolomics data from dietary melanoidins, while there have been recent reports of metabolites associated with other MRPs (69–71). As expected, protein and carbohydrates derivatives were identified as likely by-products of gut microbiome metabolism. Therefore, it can be hypothesized that melanoidins would be degraded into smaller MRPs than similar metabolites, but exploratory metabolomics studies are needed to identify microbially-derived metabolites.

In the absence of baseline knowledge about the microbial metabolism of melanoidins, most studies have focused on their effect on the microbiota. One of the earliest studies relied on *in vitro* small intestine and gut microbiota batch fermentation (72). Peptic and pancreatic digestion did not affect the model melanoidin, supporting the hypothesis that they are mostly non-digestible. On the other hand, melanoidins stimulated the overall growth of anaerobic gut bacteria, suggesting that gut microbiota members can metabolize them to gain energy (72). It was further demonstrated that native dietary melanoidins could be excreted in feces but very low amounts in urine (66, 73). These observations strengthen the hypothesis that microbiota can utilize melanoidins. Based on melanoidins physical similarity with dietary fibers (74) and their apparent fermentability by the gut microbiota, melanoidins have subsequently been proposed analogous to dietary fiber and, thereby, potential prebiotics (75). In this study, bread melanoidins were submitted to an anaerobic batch culture, and stimulation of Bifidobacteria was observed.

It should be noted that the ability of a food extract to stimulate the growth of so-called prebiotic species cannot be used as conclusive evidence of a prebiotic or even prebiotic-like effect. Indeed, when similar *in vitro* fermentation experiments were conducted with other bread melanoidins products, bifidogenic effects or the *Lactobacillus* stimulation were subject-specific and not across the board (76). The inter individuality in gut microbiota response has been commonly described (77, 78) and should not be considered as contradicting the potential dietary fiber or prebiotic effect, but it also emphasizes the need for more evidence.

In the context of barley melanoidins, it should be stated that barley dietary fiber and specifically beta-glucan, can be expected to exert beneficial prebiotic-like effects (79–81). Intriguingly, some of the initial studies on barley melanoidins have focused on their antimicrobial properties. For example, barley coffee and the melanoidin fraction specifically inhibited *Streptococcus mutans* biofilm formation (37, 38). Furthermore, potential antioxidant effects from barley and other dietary melanoidins were ascribed to the gastrointestinal tract in general and possibly mediated by the gut microbiota (33, 82, 83). More recently, we conducted an animal study to test the hypothesis of barley melanoidins acting as dietary fiber (35). We used an increasing proportion of melanoidin-rich malted barley combined with simple malted barley (0, 25, 50, 75, and 100%) fed *ad libitum* to mice over 3 weeks. Mice gut microbiota was significantly clustered by dietary treatment, and many bacterial taxa were differentially affected.

Interestingly, *Lactobacillus* responded slower to the treatment with melanoidins, while Bacteroidetes were the most responsive after 7 days. *Bifidobacterium* and *Akkermansia*, two genera considered beneficial, were stimulated by barley melanoidins, especially toward the end of the experiment. The almost immediate increase in Bacteroidetes supports the model of barley melanoidins acting like dietary fiber. However, it appears that any prebiotic-like can only be expected after a relatively long adaptation period from the gut microbiota. It can be hypothesized that this delayed effect is due to the melanoidins structure being even more complex than dietary fiber and the need for cellulose and other fermentative consortia (84, 85) to such a new energy source.

## Negative Health Impact of Melanoidins

Melanoidins have been extensively investigated for their health beneficial effects, and reports are signifying their other health beneficial properties. The beneficial health properties of food melanoidins are associated with various activities such as antioxidant, antimicrobial, anti-inflammatory, antihypertensive, and prebiotic activity (35, 86, 87). However, very few studies on the negative impacts of melanoidins on health are reported, an extensive review has been done on the impact of MRPs on nutrition and health by ALjahdali and Carbonero (61). MRPs affects the bioavailability of trace elements in rat magnesium digestion is reduced in the presence of MRPs. Nutritional quality of foods and protein digestibility is also reported to be reduced under the influence of MRPs (61, 65, 88). Diabetes, cardiovascular diseases, and even carcinogenetic effects are also associated with MRPs. High dietary carboxymethyl lysine (CML) promotes diabetes and cardiovascular diseases, whereas acrylamide inciting tumors (9, 89). Melanoidins are recalcitrant molecules and hard to biodegrade by microorganisms; industrial wastes of breweries and distilleries are rich in melanoidins, act as pollutants to the environment, and ultimately affect human health (90–92).

## Conclusion

People's food habits and living habits lead them to obesity, diabetes, hypertension, and gastrointestinal diseases, cancer, and others in the current situation. Today's need is to improve our food habits and incorporate such food materials in our daily diet to reduce the conditions mentioned above. Various studies suggested that the intake of melanoidins associated with health benefitting properties. However, melanoidins structure's chemical characteristics are sparsely known. Barley as a source of melanoidins needs more attention because barley can be grown in marginal areas with low input, used mainly in malt and brewing industries, which produces many by-products containing melanoidins. Therefore, comprehensive studies are required to understand better the chemical structure and specific health-benefiting properties of food melanoidins.

## Author Contributions

All authors listed have made a substantial, direct and intellectual contribution to the work, and approved it for publication.

## Conflict of Interest

The authors declare that the research was conducted in the absence of any commercial or financial relationships that could be construed as a potential conflict of interest.

## Publisher's Note

All claims expressed in this article are solely those of the authors and do not necessarily represent those of their affiliated organizations, or those of the publisher, the editors and the reviewers. Any product that may be evaluated in this article, or claim that may be made by its manufacturer, is not guaranteed or endorsed by the publisher.
